# Systemic Identification of* Hevea brasiliensis* EST-SSR Markers and Primer Screening

**DOI:** 10.1155/2017/6590902

**Published:** 2017-01-23

**Authors:** Benjun Hou, Suping Feng, Yaoting Wu

**Affiliations:** ^1^College of Agriculture, Hainan University, Haikou 570228, China; ^2^Institute of Cereal Research, Hainan Academy of Agricultural Sciences, Haikou 571100, China; ^3^Hainan Tropical Ocean University, Sanya 572202, China; ^4^Key Laboratory of Tropical Crop Molecular Breeding of Sanya, Sanya 572202, China

## Abstract

This research aimed to systematically identify and preliminarily validate the* Hevea brasiliensis *expressed sequence tag (EST) information using Simple Sequence Repeat (SSR) and provide evidence for further development of SSR molecular marker. The definition of general SSR features of* Hevea* EST splicing sequences and development of SSR primers founded the basis of diversity analysis and variety identification for* Hevea* tree resource. 1134 SSR loci were identified in the EST splicing sequence and distributed in 840 Unigene. The occurrence rate of SSR loci was 23.9%, and the average distribution distance of EST-SSR was 2.59 kb. The major repeat type was mononucleotide repeat motif, which accounted for 38.89%, while the corresponding value was 36.95% for dinucleotide repeat motif and 18.17% for trinucleotide repeat motif; the proportion of other motifs was only 5.99%. The superior repeat motifs for mononucleotide, dinucleotide, and trinucleotide were A/T, AG/CT, and AAG/CTT, respectively. 739 pair of primers were designed for 1134 SSR loci. PCR amplification was performed on* Hevea* Reyan5-11, Reyan87-6-47, and PR107, and 180 pairs of primers were selected which were able to amplify polymorphism bands.

## 1. Introduction

*Hevea brasiliensis*, also known as Pará rubber tree in Brazil, belonging to* Hevea* in Euphorbiaceae, is originated in the Amazon River basin, Brazil, and now is distributed in more than 40 countries and areas in Asia, Africa, Oceania, and Latin America. China is one of the major rubber producers after Indonesia, Thailand, Malaysia, and India, of which the production is the fifth largest in the world. Due to the limitation of climate condition, the* Hevea brasiliensis* planting in our country is distributed in Hainan, Guangdong, Guangxi, Yunnan, and Taiwan, among which Hainan is the main planting area.

Simple Sequence Repeat (SSR), also referred to as microsatellite DNA, short tandem (1–6) repeat sequence, is widely distributed in animal and plant gene coding region and noncoding region. SSR marker is a codominant marker, which is of good repeatability, easy operation, and wide coverage and also shows higher polymorphism compared with other markers [1, 2]. The SSR can be divided into genome SSR and expressed sequence tag-Simple Sequence Repeat (EST-SSR) according to its origin. The development of traditional genome SSR marker is time consuming, with low positive clone rate and small success probability [3–5]. Therefore, the development of SSR marker is greatly limited, and the analysis and screening usually rely on related information from closely related species. With the development of sequencing technology, the EST-related data are increasing, which enriches the EST-SSR marker. The EST-SSR not only shares the similar advantages as genome SSR marker such as high polymorphism, codominance, and good repeatability, but also possesses good generality between species [6–14].

In recent years, the development of SSR marker based on the EST data has been reported not only in fruit trees such as citrus [15], peach [16], pear [17, 18], kiwi fruit [19], walnut [20], apricot [21], and lychee [22, 23], but also in those Euphorbiaceae family trees such as cassava [24, 25], castor oil [26],* Jatropha* [27], and* Vernicia* [28, 29]. SSR marker has been widely used in variety identification and improvement, resource analysis, genetic map construction, functional gene discovery, and so forth. So far, Hua et al. [30], An et al. [31], Li et al. [32], and Feng et al. [33] have reported the* Hevea* EST-SSR markers, but the EST they used were ones before 2009. EST data was developed very rapidly, and more than 20,000 EST sequences were developed from cDNA gene bank in the last two years. So the redevelopment of EST is very important.

In this study, we designed SSR loci specific primer according to the data of 38815* Hevea* EST sequences included in NCBI database before June 1, 2012, performed polymorphism test on synthetic primers, and developed* Hevea* EST-SSR molecular marker. This study aimed to provide evidence for further development of SSR molecular marker and lay foundation for diversity analysis and variety identification of* Hevea* tree resource.

## 2. Materials and Methods

### 2.1. Plant Material and EST Retrieval

Fresh leaf samples were collected from Reyan5-11, Reyan87-6-47, and PR107 of the cultivated rubber tree species (*Hevea brasiliensis*) growing in the Rubber Cultivation Research Institute, Chinese Academy of Tropic Agricultural Science (Danzhou). Leaf genomic DNA was extracted following the one-step method from Bioteke Co., Ltd.

EST sequences were obtained via the ENTREZ search tool of the EST database at the NCBI (http://www.ncbi.nlm.nih.gov). A total of 38,815 ESTs available on June 1, 2012, were obtained for this study.

The Seqclean software (https://sourceforge.net/projects/seqclean/) was used to remove the polyA/polyT tail, clip low-quality ends (the ends rich in undetermined bases), and trash the ones which are too short (shorter than 100 bp) or which appear to be sequence (mostly low-complexity sequence, vectors, adapters, mitochondrial, ribosomal, bacterial, and other species than the target organism, etc.).

CD-HIT program (http://weizhongli-lab.org/cd-hit/) was used for clustering the ESTs, and then the same ESTs were removed.

The CAP3 (http://seq.cs.iastate.edu/cap3.html) was used to assemble EST sequence.

### 2.2. Data Mining

After pretreatment, the MISA software (http://pgrc.ipk-gatersleben.de/misa/) was used to search for SSRs from the rubber ESTs. The search criteria were mononucleotide repeats ≥10, dinucleotide repeats ≥6, trinucleotide repeats ≥5, and tetranucleotide to hexanucleotide ≥4; meanwhile, those interrupted composite SSRs had also been selected (interval bases ≤100). Dinucleotide repeats such as AT/TA and CT/GA were treated as the same type of repeat motif.

The SSR-ESTs were used to design primers with primer3 (http://sourceforge.net/projects/primer3/files/primer3) where the main parameters were GC content of 20%–80%, annealing temperature (*T*_m_) of 50–63°C, and expected amplified products size of 100–300 bp. All primers were synthesized by the Beijing Dingguo Changsheng Biotechnology Co., Ltd. EST-SSRs were amplified using the Whatman Biometra T1 Thermocycler. Each PCR reaction consisted of 2 ul of 10x buffer, 0.25 ul of 10 M dNTP, and 1 ul each of forward and reverse primer (20 umol), 0.15 ul of Taq polymerase (5 U/ul) (Shanghai Sangon Biological Engineering Technology & Services Co., Ltd.). The PCR reaction profile was predenaturation at 94°C for 2 min followed by 30 cycles of 94°C for 30 s, 55–60°C for 45 s and 72°C for 1 min, and finally 72°C for an extension of 5 min.

## 3. Results and Analysis

### 3.1. Development Process of* Hevea* EST-SSR Marker

**Figure 1:**
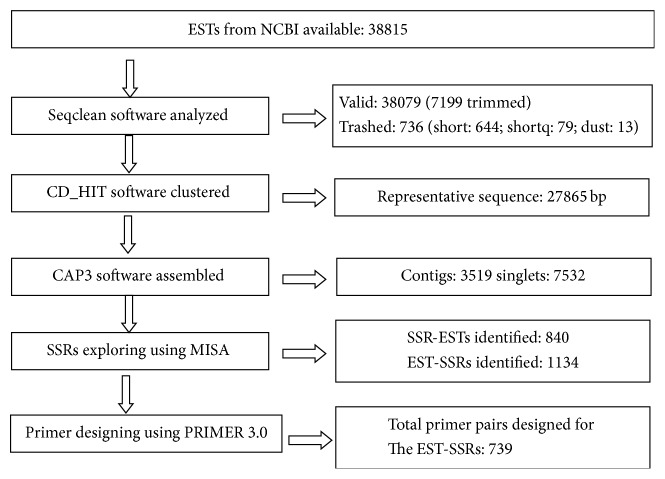
Scheme used for data exploring and development of EST-SSRs markers from* Hevea brasiliensis* ESTs.

The development process of SSR was shown in Figure 1. The data of 38815 EST sequences of* Hevea brasiliensis* in the FASTA format were downloaded from NCBI dbEST database (up to June 1, 2012). 38079 sequences were obtained, and 736 were trashed. Among the 736 sequences, 644 were short, 79 were shortq, and 13 were dust. CD_HIT software was adopted for cluster, and redundant sequences were removed, thus obtaining 27865 sequences. The redundancy rate reached 28.21%. After splicing was performed on the 27865 sequences with CAP3 software, 3519 splicing sequences and 7532 single sequences were obtained. SSR loci were searched in the 3519 sequences using MISA software, and 1134 loci were obtained. 739 pairs of primers were designed using primer3 software for the 1134 SSR loci.

### 3.2. The Occurrence Rate and Distribution of* Hevea* SSR Loci

Using MISA software, SSRs loci were searched on the 3519 splicing sequences composed of EST sequences and possessing a total length of 2942162 bp, and the results showed that there were 840 sequences containing SSR repeat loci sequences in 3519 splicing sequences. The occurrence rate of SSR was 23.9% (the ratio of the amount of SSR-contained Unigene to the total amount of Unigene), as shown in Table 1.

The total length of 840 SSR sequences was 798917 bp, and the analysis on distribution features of SSRs-contained sequences discovered that the quantity of Unigene with single SSR locus was 620 (73.81%), while that of Unigene with two or more SSR loci was 220 (26.19%), and that of Unigene with compound type of SSR loci was 136, 1134 SSRs in total. From the perspective of distribution, the distribution rate (ratio of SSR quantity to total Unigene quantity) was 32.2%. The average distance of SSR was 2.59 kb (ratio of total length of Unigene to SSR quantity); namely, one EST-SSR occurred every 2.59 kb. The occurrence rate and distribution rate of SSR loci of* Hevea* were quite high.

### 3.3. The Repeat Types and Proportion of SSR Loci

From Table 2, the statistical analysis on repeat motifs of all SSR loci showed that, in the* Hevea* EST-SSR, the type of repeat nucleotide was 1 to 6, and the occurrence rate of different SSR types was different. The quantity of mononucleotide repeat sequence motifs was the largest, 441 in total, accounting for 38.89%; the second largest quantity of repeat sequence motifs was dinucleotide, 419 in total, accounting for 36.95%, and the quantity of trinucleotide repeat sequence motifs was 206 and accounted for 18.17%. The total ratio of mono-, di-, and trinucleotide was 94.01%. The rest of nucleotide repeat motifs accounted for 5.99%. Among them, the quantity of tetranucleotide, pentanucleotide, and hexanucleotide motifs was 33, 9, and 26, respectively, which accounted for 2.91%, 0.79%, and 2.29%, respectively.

The results above indicated that the major repeat sequence type of* Hevea* SSR loci was mononucleotide repeat, and the dinucleotide and trinucleotide repeats were common in the polynucleotide repeat. The quantity of different SSR repeat types showed a decline trend as the quantity of motif nucleotide increased.

### 3.4. The Repeat Motif Type and Times of Repetition

#### 3.4.1. The Type of SSR Repeat Motif

The different types of* Hevea* EST-SSR repeats possessed various kinds of motifs, and 55 types of repeat sequence motifs were detected in SSRs loci, including 2 kinds of mononucleotide repeat motifs, 3 kinds of dinucleotide repeat motifs, 10 kinds of trinucleotide repeat motifs, 12 kinds of tetranucleotide, 5 kinds of pentanucleotide, and 23 kinds of hexanucleotide repeat motifs.

#### 3.4.2. The Times of SSR Repeat Motif Repetition

From Table 3, we could see that the number of times of SSR motifs repetition of* Hevea* splicing sequences ranged from 4 to 78 (those >15 times were not listed in the table). 834 SSR motifs showed 4 to 15 repetitions, accounting for 73.54%, and 300 SSR motifs had repetitions >15, accounting for 26.46%. The repetition number of times ranked first was 10 in 154 (13.58%), followed by 5 in 114 (10.05%), 6 in 112 (9.88%), 11 in 92 (8.11%), and 12 in 63 (5.56%). The first 5 repetition numbers of times were among the range of 4 to 15.

The repetition number of times was the maximum in the minimum searching criteria of each repetition type except AAAT/ATTT (the maximum repetition number of times was 5). The common types of nucleotide motifs in those with repetition number of times >15 (not listed in the table) were mononucleotide, dinucleotide, and trinucleotide. The repetition numbers of times were 78 for A/T, 19 for C/G, 18 for AC/GT, 50 for AG/CT, 39 for AT/AT, 17 for AAG/CTT, and 16 for AAT/ATT.

#### 3.4.3. The Repetition Number of Times of Each Type of SSR Repeat Motifs

**Figure 2:**
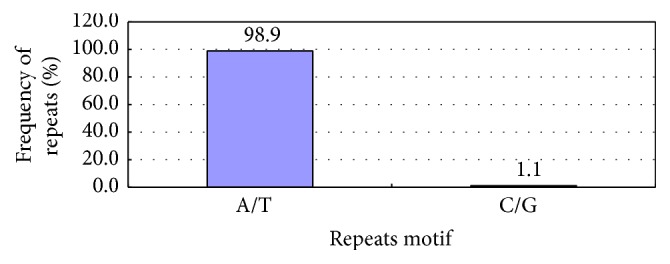
Mononucleotide repeats of Unigene.

From the types of SSR motifs and occurrence rate (Table 3, Figure 2), there were 2 types of mononucleotide repeats, that is, (A/T)*n* and (C/G)*n*, primarily (A/T)*n*, which accounted for 98.9% of the repeat motifs. (A/T)*n* repeat ranged from 10 to more than 15, among which 11 is the most common, followed by 12. For (C/G)*n*, 10 repetitions occurred 4 times, and >15 repetitions presented only once.

**Figure 3:**
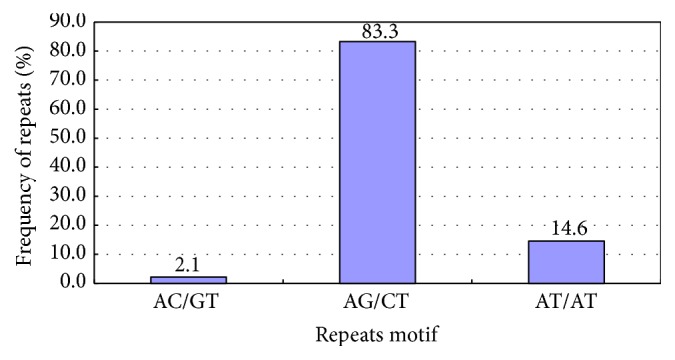
Dinucleotide repeats of Unigene.

There were 3 types of dinucleotide repeats (Table 3, Figure 3), that is, (AC/GT)*n*, (AG/CT)*n*, and (AT/AT)*n*, wherein (AG/CT)*n* was present most frequently, accounting for 83.3%, followed by (AT/AT)*n*, accounting for 14.6%. The number of times of (AG/CT)*n* repeat ranged from 6 to more than 15, among which 10 was the most, followed by 11.

**Figure 4:**
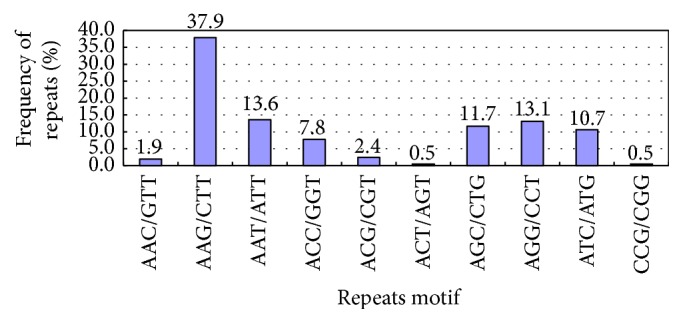
Trinucleotide repeats of Unigene.

There were 10 types of trinucleotide repeats (Table 3, Figure 4), primarily (AAG/CTT)*n*, (AAT/ATT)*n*, (AGC/CTG)*n*, (AGG/CCT)*n*, and (ATC/ATG)*n*, which accounted for 37.9%, 13.6%, 11.7%, 13.1%, and 10.7%, respectively. Except (AAG/CTT)*n* repeating >15 times occurred once and (AAT/ATT)*n* repeating more than 15 times occurred twice, all the other repeat types show less than 13 repetitions. (ACT/AGT)*n* occurred once at 6 repetitions, and (CCG/CGG)*n* was present once at 5 repetitions.

**Figure 5:**
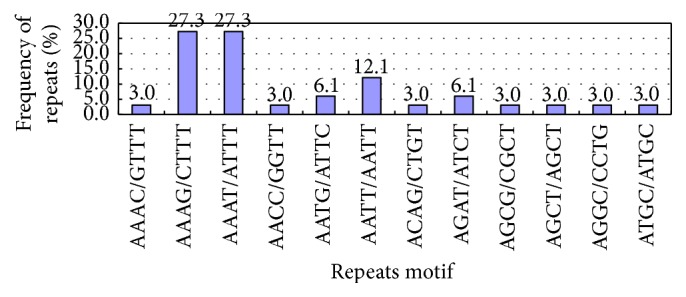
Tetranucleotide repeats of Unigene.

There were 12 types of tetranucleotide repeats (Table 3, Figure 5), primarily (AAAG/CTTT)*n*, (AAAT/ATTT)*n*, and (AATT/AATT)*n*, which accounted for 27.3%, 27.3%, and 12.1%, respectively. Such repeat types had usually 4–6 repetitions. (AAAG/CTTT)*n* occurred once at 6 repetitions, once at 5, and 7 times at 4. (AAAT/ATTT)*n* was present twice at 6 repetitions, once at 5, and 6 times at 4. (AAAC/GTTT)*n* and (AGAT/ATCT)*n* occurred once at 5 repetitions, and other repeat types occurred at 4 repetitions.

**Figure 6:**
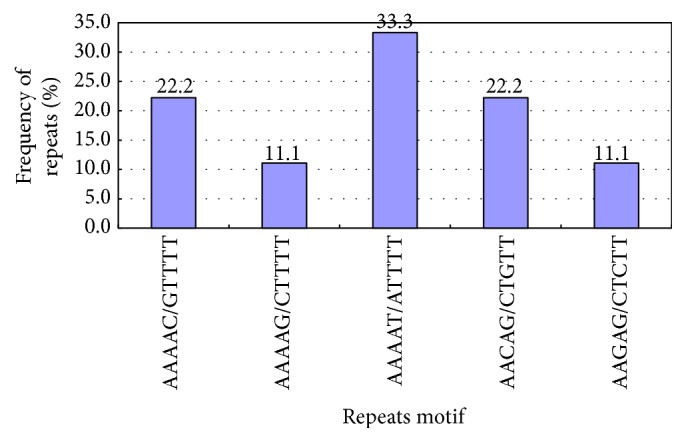
Pentanucleotide repeats of Unigene.

There were 5 types of pentanucleotide repeats (Table 3, Figure 6), that is, (AAAAC/GTTTT)*n*, (AAAAG/CTTTT)*n*, (AAAAT/ATTTT)*n*, (AACAG/CTGTT)*n*, and (AAGAG/CTCTT)*n*, which accounted for 22.2%, 11.1%, 33.3%, 22.2%, and 11.1%, respectively. Such repeat types occurred usually 4-5 times. (AAAAT/ATTTT)*n* occurred twice at 5 repetitions, once at 4. The other repeat types occurred at 4 repetitions.

**Figure 7:**
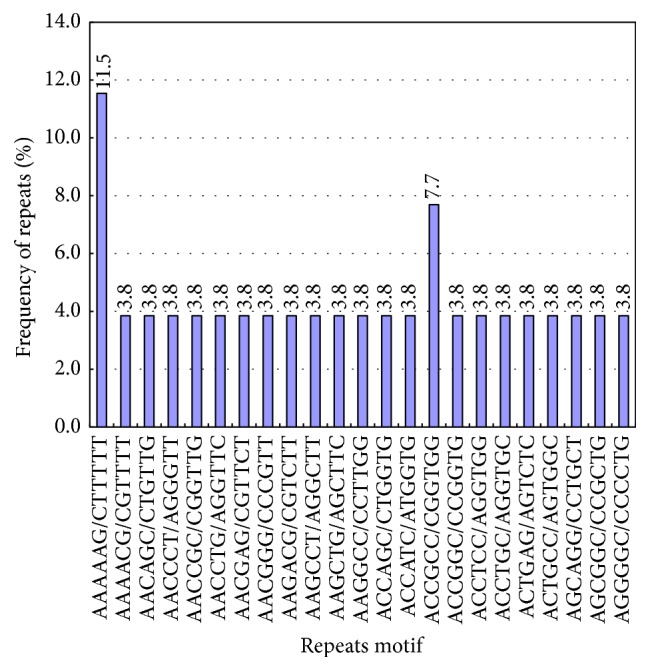
Hexanucleotide repeats of Unigene.

There were 23 types of hexanucleotide repeats (Table 3, Figure 7), primarily (AAAAAG/CTTTTT)*n* and (ACCGCC/CGGTGG)*n*, which accounted for 11.5% and 7.7%. The rest accounted for 3.8%. Such repeat types usually occurred 4–10 times.

### 3.5. Distribution Characteristics of SSR Loci Length and Sequence Length in* Hevea brasiliensis*

The distribution characteristics of SSR locus length of 840 simple repeat sequences (SSR) in* Hevea brasiliensis* were shown in Tables 2 and 4. SSR length ranged from 10 to 100 bp, 24859 bp in total and 21.92 bp on average. SSR length was less than 30 bp mostly, with 953 repetitive sequence motifs, accounting for 84.04% of the total. Among them, there were 763 repetitive sequence motifs of 10–20 bp (67.28%) and 190 repetitive sequence motifs of 21–30 bp (16.76%). The number of SSRs with the length >30 bp was 181, accounting for 15.96%. The number of repetitive sequence motifs with the length of 31–40 bp, 41–50 bp, 51–60 bp, 61–70 bp, 71–80 bp, 81–90 bp, and 91–100 bp was 66, 39, 31, 21, 11, 7, and 6, respectively, with the corresponding proportion of 5.82%, 3.44%, 2.73%, 1.85%, 0.97%, 0.62%, and 0.53%. For SSR loci of mono-, di-, tri-, tetra-, penta-, and hexanucleotide, the total length of base was 6249, 13022, 4044, 568, 2944, and 786 bp, respectively; the average length of different SSR loci was 14.17, 31.08, 19.63, 17.21, 21.11, and 30.23 bp, respectively, and the microsatellite contained in splicing sequence had a great difference in length.

The length distribution of 840 Simple Sequence Repeats (SSRs) in* Hevea brasiliensis* was shown in Table 5. The length ranged from 146 to 3194 bp, 798917 bp in total, and 951.09 bp on average. 55 SSR had the length less than 500 bp, accounting for 6.55%; the majority (695) had the length from 501 to 1500 bp, accounting for 82.74%; 60 showed the length of 1501–2000 bp, accounting for 7.14%; and 30 exhibited longer sequence, accounting for 3.57%.

### 3.6. Processing Results Using PRIMER3.0 Software

PRIMER3.0 software was used to design primers for 840 sequences. Among 1134 SSR loci in* Hevea brasiliensis*, 998 SSR loci were qualified for primer design, and 136 were unqualified. In 998 SSR loci, primers were successfully designed for 739 loci, with the success rate of 74.05%, and primers were not successfully designed for 259 loci. Among the 258 sequences corresponding to the 259 loci, each of 203 sequences had one SSR loci, and primers were not successfully designed since the SSR loci were located at the end or in the front of the sequence, while each of 48 sequences possessed two SSR loci, and primers were not obtained for one of the two loci on each sequence; Contig1648 splicing sequence had 2 SSR loci, and primers were not successfully designed for both loci. In 6 splicing sequences each had three SSR loci, and primers were not obtained for one of the three loci on each sequence.

### 3.7. Preliminary Screening of SSR Primers

The 739 pairs of synthetic primers were amplified and screened with Reyan5-11, PR107, and Reyan87-6-47 genomic DNA in* Hevea brasiliensis* as the template, and electrophoresis results showed that there were a total of 180 pairs of polymorphic primers, accounting for 24.36%; 386 pairs showed clear bands but were nonpolymorphic, accounting for 52.23%; and 173 pairs showed no amplified band.

## 4. Discussion

### 4.1. Occurrence Rate of SSR Loci

As the rapid development of the research in plant functional genomics, the ESTs of public database are showing an exponential growth trend. It is becoming a locus to develop new EST-SSR marks by searching the SSR loci of EST sequences. In this study, we found 840 sequences containing 1,134 SSR loci from 38,815 EST sequences of* Hevea brasiliensis* searched in the public database and the occurrence and distribution rate were 23.9% and 32.2%, respectively. It meant that one EST-SSR was present every 2.59 kb on average, which was higher than that (9.1%) of 11809 ESRs in* Hevea brasiliensis* reported by Hu et al. [30] before May 10, 2007, and that (11.42%) in* Hevea brasiliensis* by An et al. [31]. Its distribution distance was higher than 3.93 kb reported by An et al. [31] and 3.96 kb by Li et al. [32] but lower than 2.25 kb by Feng et al. [33] and 281.39 bp by Li et al. [34]. All above are related to testing standard, data size, and test instrument.

Compared with other plants in* Hevea*, its SSR's rate of occurrence was higher than that of castor (27.94%) [26] and cassava (11.02%) [25]; and the distance of distribution was higher than that of castor (2.81 kb) [21] and cassava (6.02 kb) [26], but lower than that of* Vernicia fordii* (2.25 kb) [29].

The occurrence rate of SSR was higher than that in oil palm [35] (6.13%), casuarina [36] (2.93%), and poplar [37] (14.83%). And the distribution distance of was longer than that in casuarina [36] (19.83 kb),* Ginkgo biloba* [38] (12.02 kb),* Camellia sinensis* [39] (3.68 kb), and citrus [40] (5.7 kb).

There is a big difference in the occurrence and distribution rate of EST-SSR between different plants or within the same species, which may be related to varied genome constitution of plants, transcriptome sequencing methods, quantity of data, and microsatellite search criteria [41].

### 4.2. SSR Repetition Types

SSR repetition type has different distributions in different plants. In this study, there were 1 to 6 SSR types. The mononucleotide repeat had the maximum value, accounting for 38.89% and mainly involving (A/T)*n* (accounting for 98.9% of its repeat motifs). Although the polyT and polyA sequence at the 5′- and 3′-end were removed during the pretreatment for the original sequence, the A/T type still had a high proportion of 98.9% in the total mononucleotide SSR, which indicated presence of false positive A/T. However, the polynucleotide was mainly involved in dinucleotide and trinucleotide repeats, which was consistent with the result of Hu et al. [30], An et al. [31], Li et al. [32], and Feng et al. [33]. Dinucleotide and trinucleotide repeats accounted for 36.95% and 18.17% of the total SSR, respectively; in dinucleotide and trinucleotide repeat, (AG/CT)*n* and (AAG/CTT)*n* were in the majority, respectively, accounting for 83.3% and 37.9% of the repeat motifs.

In a study of castor, Zhou et al. [26] found that the mononucleotide repeat type had the highest occurrence rate (37.51%) among 1–6 bp repeat motifs, followed by trinucleotide repeat type (34.63%) and dinucleotide repeat type (25.61%).

Peng et al. [25] found that dinucleotide repeat type had the highest occurrence rate (36.03%) among 1–6 bp repeat motifs in cassava, followed by trinucleotide (31.84%) and mononucleotide repeat type (30.10%). In terms of repeat motifs, A/T ranked first (29.23%) and AG/CT second (24.75%).

Through investigation of EST sequences of* Vernicia fordii*, Jia et al. [28] and Xu et al. [29] found that dinucleotide and trinucleotide repeats were common. For dinucleotide repeat, AG/CT shared the largest proportion; and for trinucleotide repeat, AAG/CTT showed the largest proportion.

Among the nucleotide repeat types, A/T was the most abundant repeat motif, which may be related to the energy in the base. As less energy was required to break AT bond compared with GC bond, AT fluctuated more easily. A/T bonds were more common in SSR, indicating that A/T motif-rich SSR types may be more commonly seen in plants [42, 43].

### 4.3. SSR Repeat Lengths

The usability of SSR molecular markers significantly depends on its polymorphism, which is mainly influenced by the length of SSR. It is generally considered that the variation of SSR lengths mainly results from sliding and mismatching of DNA chains during duplication and DNA repair, or the unequal sister chromatid exchanges (SCEs) during mitosis or meiosis. In this study, the SSR length of* Hevea brasiliensis* varied from 10 bp to 100 bp, 24859 bp in total, and 21.92 bp on average. In a study exploring variation of SSR lengths in Eucalyptus, Li et al. [44] found that SSR repeat motif lengths were negatively correlated with the variation rate of EST-SSR lengths, as well as the abundance of SSR loci, with the SSR lengths ranging from 16 to 64 bp and 18.5 bp on average.

### 4.4. Primer Amplification

In this study, 173 out of 739 pairs of primers cannot be effectively amplified. It may be caused by the following reasons: first, the sequence amplified by primer pairs contains large introns, which cannot be displayed on electrophoresis. Second, one or both ends of the primer pairs happen to be located at a certain shear site. Third, EST fragments change. Fourth, the annealing temperature is too high or too low.

## 5. Conclusions

In the* Hevea brasiliensis* EST splicing sequences, the occurrence rate of SSR loci is 23.9%, and an EST-SSR is present every 2.59 kb on average. The SSR repeat type is primarily mononucleotide repeat motifs, accounting for 38.89%; the dinucleotide and trinucleotide repeat sequence motifs share the proportions of 36.95% and 18.17%, respectively; other motifs only accounted for 5.99%. A/T, AG/CT, and AAG/CTT are the superior repeat motifs of mononucleotide, dinucleotide, and trinucleotide. 739 pairs of primers are designed successfully for 1134 SSR loci. Through PCR amplification for Reyan5-11, Reyan87-6-47, and PR107 of* Hevea brasiliensis*, 180 pairs of primers that can show polymorphic bands are selected. As EST is an effective source for developing SSR marker, it is feasible and effective to develop SSR markers from EST in* Hevea brasiliensis*.

## Figures and Tables

**Table 1:** Results of microsatellite search.

Designation	Numbers
Total number of sequences examined	3519
Total size of examined sequences (bp)	2942162
Number of SSR containing sequences	840
Number of sequences containing 1 SSR	620
Number of sequences containing more than 1 SSR	220
Number of SSRs present in compound formation	136
Total number of identified SSRs	1134

**Table 2:** Distribution to different repeat type classes.

Motifs type	SSR Numbers	Accounting for	SSR Length	SSR average length
Mononucleotide	441	38.89	6249	14.17
Dinucleotide	419	36.95	13022	31.08
Trinucleotide	206	18.17	4044	19.63
Tetranucleotide	33	2.91	568	17.21
Pentanucleotide	9	0.79	190	21.11
Hexanucleotide	26	2.29	786	30.23
Total	1134	100	24859	21.92

**Table 3:** Frequency of classified repeat types (considering sequence complementary) in the analysed 840 splicing sequences.

Repeats motif	Number of repeat units													Total repeats	Frequency of repeats
	4	5	6	7	8	9	10	11	12	13	14	15	>15		
A/T	—	—	—	—	—	—	119	66	45	28	23	18	137	436	38.45
C/G	—	—	—	—	—	—	4	—	—	—	—	—	1	5	0.44
AC/GT	—	—	3	2	2	—	—	—	1	—	—	—	1	9	0.79
AG/CT	—	—	52	22	25	23	20	16	15	9	13	13	141	349	30.78
AT/AT	—	—	15	6	5	4	2	3	1	4	4	—	17	61	5.38
AAC/GTT	—	2	1	—	—	—	—	1	—	—	—	—	—	4	0.35
AAG/CTT	—	28	15	6	9	11	4	3	—	1	—	—	1	78	6.88
AAT/ATT	—	15	5	1	2	—	—	2		1	—	—	2	28	2.47
ACC/GGT	—	9	2	2	1	—	2	—	—	—	—	—	—	16	1.41
ACG/CGT	—	3	—	—	—	—	1	1	—	—	—	—	—	5	0.44
ACT/AGT	—	—	1	—	—	—	—	—	—	—	—	—	—	1	0.09
AGC/CTG	—	14	6	2	2	—	—	—	—	—	—	—	—	24	2.12
AGG/CCT	—	16	4	6	—	1	—	—	—	—	—	—	—	27	2.38
ATC/ATG	—	10	4	3	3	—	1	—	1	—	—	—	—	22	1.94
CCG/CGG	—	1	—	—	—	—	—	—	—	—	—	—	—	1	0.09
AAAC/GTTT		1	—	—	—	—	—	—	—	—	—	—	—	1	0.09
AAAG/CTTT	7	1	1	—	—	—	—	—	—	—	—	—	—	9	0.79
AAAT/ATTT	6	1	2	—	—	—	—	—	—	—	—	—	—	9	0.79
AACC/GGTT	1	—	—	—	—	—	—	—	—	—	—	—	—	1	0.09
AATG/ATTC	2	—	—	—	—	—	—	—	—	—	—	—	—	2	0.18
AATT/AATT	4	—	—	—	—	—	—	—	—	—	—	—	—	4	0.35
ACAG/CTGT	1	—	—	—	—	—	—	—	—	—	—	—	—	1	0.09
AGAT/ATCT	1	1	—	—	—	—	—	—	—	—	—	—	—	2	0.18
AGCG/CGCT	1	—	—	—	—	—	—	—	—	—	—	—	—	1	0.09
AGCT/AGCT	1	—	—	—	—	—	—	—	—	—	—	—	—	1	0.09
AGGC/CCTG	1	—	—	—	—	—	—	—	—	—	—	—	—	1	0.09
ATGC/ATGC	1	—	—	—	—	—	—	—	—	—	—	—	—	1	0.09
AAAAC/GTTTT	2	—	—	—	—	—	—	—	—	—	—	—	—	2	0.18
AAAAG/CTTTT	1	—	—	—	—	—	—	—	—	—	—	—	—	1	0.09
AAAAT/ATTTT	1	2	—	—	—	—	—	—	—	—	—	—	—	3	0.26
AACAG/CTGTT	2	—	—	—	—	—	—	—	—	—	—	—	—	2	0.18
AAGAG/CTCTT	1	—	—	—	—	—	—	—	—	—	—	—	—	1	0.09
AAAAAG/CTTTTT	3	—	—	—	—	—	—	—	—	—	—	—	—	3	0.26
AAAACG/CGTTTT	1	—	—	—	—	—	—	—	—	—	—	—	—	1	0.09
AACAGC/CTGTTG	—	1	—	—	—	—	—	—	—	—	—	—	—	1	0.09
AACCCT/AGGGTT	1	—	—	—	—	—	—	—	—	—	—	—	—	1	0.09
AACCGC/CGGTTG	—	1	—	—	—	—	—	—	—	—	—	—	—	1	0.09
AACCTG/AGGTTC	—	1	—	—	—	—	—	—	—	—	—	—	—	1	0.09
AACGAG/CGTTCT	—	1	—	—	—	—	—	—	—	—	—	—	—	1	0.09
AACGGG/CCCGTT	—	1	—	—	—	—	—	—	—	—	—	—	—	1	0.09
AAGACG/CGTCTT	—	1	—	—	—	—	—	—	—	—	—	—	—	1	0.09
AAGCCT/AGGCTT	1	—	—	—	—	—	—	—	—	—	—	—	—	1	0.09
AAGCTG/AGCTTC	1	—	—	—	—	—	—	—	—	—	—	—	—	1	0.09
AAGGCC/CCTTGG	1	—	—	—	—	—	—	—	—	—	—	—	—	1	0.09
ACCAGC/CTGGTG	—	—	—	—	—	—	1	—	—	—	—	—	—	1	0.09
ACCATC/ATGGTG	—	—	1	—	—	—	—	—	—	—	—	—	—	1	0.09
ACCGCC/CGGTGG	2	—	—	—	—	—	—	—	—	—	—	—	—	2	0.18
ACCGGC/CCGGTG	—	1	—	—	—	—	—	—	—	—	—	—	—	1	0.09
ACCTCC/AGGTGG	1	—	—	—	—	—	—	—	—	—	—	—	—	1	0.09
ACCTGC/AGGTGC	—	—	—	—	—	1	—	—	—	—	—	—	—	1	0.09
ACTGAG/AGTCTC	—	1	—	—	—	—	—	—	—	—	—	—	—	1	0.09
ACTGCC/AGTGGC	—	1	—	—	—	—	—	—	—	—	—	—	—	1	0.09
AGCAGG/CCTGCT	—	—	—	—	1	—	—	—	—	—	—	—	—	1	0.09
AGCGGC/CCGCTG	—	1	—	—	—	—	—	—	—	—	—	—	—	1	0.09
AGGGGC/CCCCTG	1	—	—	—	—	—	—	—	—	—	—	—	—	1	0.09
Total	45	114	112	50	50	40	154	92	63	43	40	31	300	1134	100
Account for (100%)	3.97	10.05	9.88	4.41	4.41	3.53	13.58	8.11	5.56	3.79	3.53	2.73	26.46		

**Table 4:** SSR locus length.

SSR locus length (bp)	Number of SSRs	Accounting for (100%)
10–20	763	67.28
21–30	190	16.76
31–40	66	5.82
41–50	39	3.44
51–60	31	2.73
61–70	21	1.85
71–80	11	0.97
81–90	7	0.62
91–100	6	0.53

**Table 5:** Sequence length of 840.

Sequence length	Sequence numbers	Accounting for
146–500	55	6.55
501–1000	503	59.88
1001–1500	192	22.86
1501–2000	60	7.14
2001–2500	23	2.74
2501–3000	6	0.71
more than 3000	1	0.12
